# Role of CX3CL1/CX3CR1 Signaling Axis Activity in Osteoporosis

**DOI:** 10.1155/2019/7570452

**Published:** 2019-11-12

**Authors:** Piotr Wojdasiewicz, Pawel Turczyn, Barbara Dobies-Krzesniak, Justyna Frasunska, Beata Tarnacka

**Affiliations:** ^1^Department of Rehabilitation, Eleonora Reicher National Institute of Geriatrics, Rheumatology and Rehabilitation, Spartańska 1, 02-637 Warsaw, Poland; ^2^Department of General and Experimental Pathology, Centre for Preclinical Research and Technology (CePT), Medical University of Warsaw, Pawińskiego 3C, 02-106 Warsaw, Poland; ^3^Department of Rehabilitation, Warsaw Medical University, 1st Faculty of Medicine, Spartańska 1, 02-637 Warsaw, Poland

## Abstract

Osteoporosis is a civilization disease which is still challenging for contemporary medicine in terms of treatment and prophylaxis. It results from excessive activation of the osteoclastic cell line and immune cells like macrophages and lymphocytes. Cell-to-cell inflammatory information transfer occurs via factors including cytokines which form a complex network of cell humoral correlation, called cytokine network. Recently conducted studies revealed the participation of CX3CL1 chemokine in the pathogenesis of osteoporosis. CX3CL1 and its receptor CX3CR1 present unique properties among over 50 described chemokines. Apart from its chemotactic activity, CX3CL1 is the only chemokine which may function as an adhesion molecule which facilitates easier penetration of immune system cells through the vascular endothelium to the area of inflammation. The present study, based on world literature review, sums and describes convincing evidences of a significant role of the CX3CL1/CX3CR1 axis in processes leading to bone mineral density (BMD) reduction. The CX3CL1/CX3CR1 axis plays a principal role in osteoclast maturation and binding them with immune cells to the surface of the bone tissue. It promotes the development of inflammation and production of many inflammatory cytokines near the bone surface (i.e., TNF-*α*, IL-1*β*, and IL-6). High concentrations of CX3CL1 in serum are directly proportional to increased concentrations of bone turnover and inflammatory factors in human blood serum (TRACP-5b, NTx, IL-1*β*, and IL-6). Regarding the fact that acting against the CX3CL1/CX3CR1 axis is a potential target of immune treatment in osteoporosis, the number of available papers tackling the topic is certainly insufficient. Therefore, it seems justified to continue research which would precisely determine its role in the metabolism of the bone tissue as one of the most promising targets in osteoporosis therapy.

## 1. Introduction

**Figure 1:**
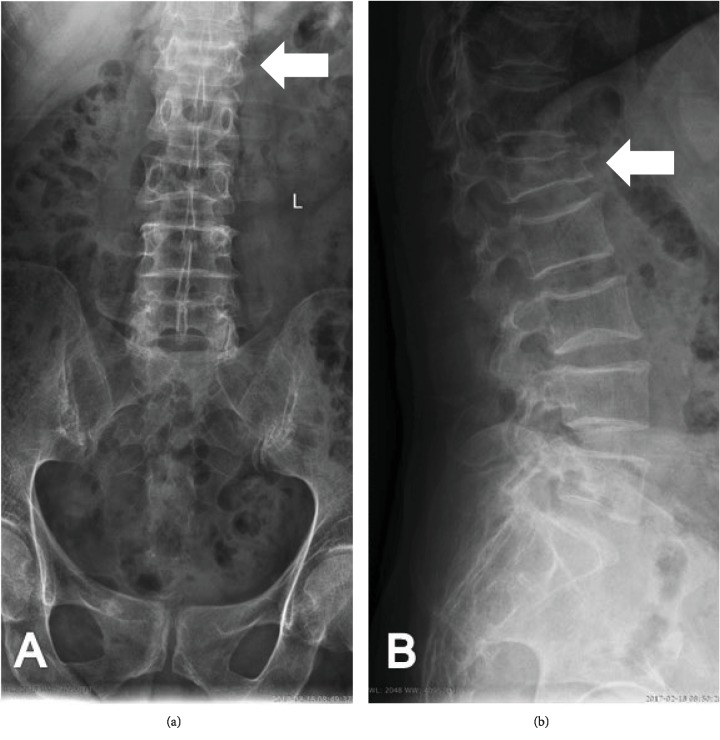
Radiographs showing the advanced osteoporosis with pathological fracture of the first lumbar vertebra (L1) in anteroposterior (a) and lateral (b) view. White arrows indicate the fracture site.

Osteoporosis results from the disturbance of balance between the development of bone tissue and its resorption. The phenomena are induced by two types of cells: osteoblasts with their anabolic properties and osteoclasts responsible for osseous tissue catabolism [1, 2]. Excessive activation of the osteoclastic cell line is induced via an increased inflammatory activity of numerous cell types—macrophages, lymphocytes, fibroblasts, osteoblasts, and osteoclasts (autocrine and paracrine mechanism) at the cellular level [3]. Cell-to-cell inflammatory information transfer occurs via factors including inflammatory cytokines which form a complex network of cell humoral correlation, called “cytokine network,” with anti-inflammatory cytokines, growth factors, and other molecules [4]. As regards the pathophysiology of osteoporosis, extensive research has been documented for the catabolic activity of numerous inflammatory cytokines, including IL-1*β* (interleukin 1 beta), TNF-*α* (tumor necrosis factor *α*), IL-6 (interleukin 6), IL-12 (interleukin 12), and IL-17 (interleukin 17), on bone mineral density (BMD) [5]. Research into the causes and optimal treatment of this disease still seems to be a necessary and demanding task due to an increasing percentage of osteoporotic patients (longer lifespan, deteriorating environmental conditions, and medications) and many health complications, e.g., osteoporotic fractures (Figure 1).

Recently conducted studies revealed the participation of CX3CL1 chemokine (CX3C motif chemokine ligand 1, fractalkine) in the pathogenesis of osteoporosis [6, 7]. Chemokines belong to the cytokine group. They are low molecular weight proteins participating in regulating the migration of leukocytes and other cells influencing inflammatory process [8]. CX3CL1 and CX3CR1 (CX3C chemokine receptor 1) present unique properties among over 50 described chemokines. Apart from its chemotactic activity, CX3CL1 is the only chemokine which has a different molecular structure and may function as an adhesion molecule which facilitates easier penetration of immune system cells through the vascular endothelium to the site of inflammation [9, 10]. Few studies are available worldwide which tackle the issue of laboratory evaluation of the role of CX3CL1 in the pathophysiology of osteoporosis. Therefore, it is justified to organize and critically assess previous findings regarding the subject, especially in the context of possible new osteoporosis therapies.

## 2. Structure and Function of the CX3CL1/CX3CR1 Signaling Axis

**Figure 2:**
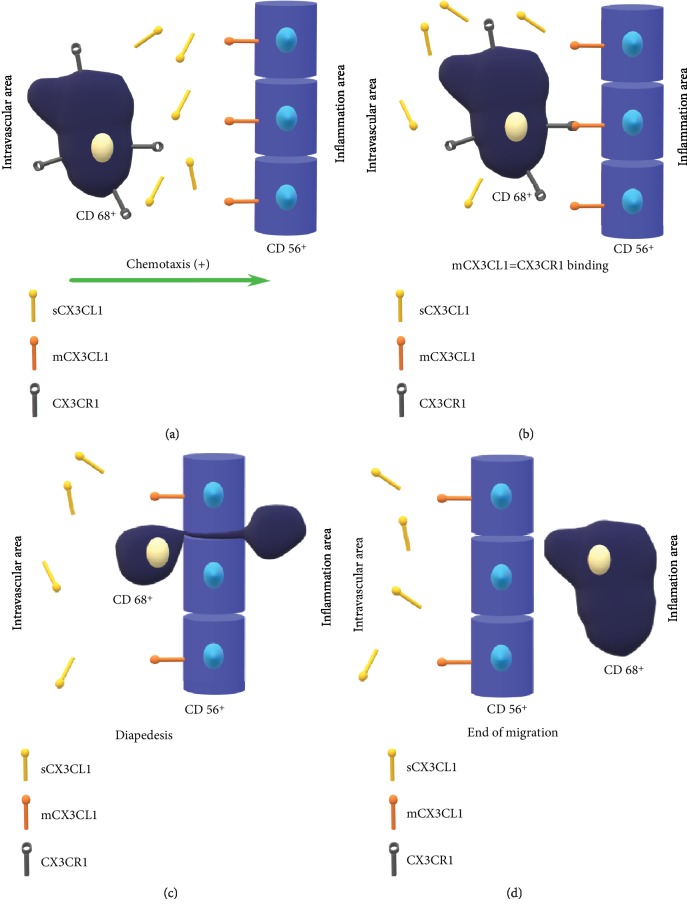
Schematic diagram showing the migration process of CX3CR1^+^ cells (in this case CD68^+^ cell) from the intravascular area via the vascular endothelium (CD56^+^ cells) to the site of inflammation. The chemotaxis process is initiated by the increasing concentration of sCX3CL1 (a). CD68^+^ cell moves towards endothelium cells that present mCX3CL1 on their surface (b). Binding of the CX3CR1 to mCX3CL1 begins the reaction cycle allowing the CD68^+^ to start the diapedesis process (c). The end of the migration process occurs when CD68^+^ is outside of the blood vessel (d). CX3CR1: CX3C chemokine receptor 1; CD: cluster of differentiation; sCX3CL1: soluble form of CX3CL1; mCX3CL1: membrane-anchored form of CX3CL1.

CX3CL1 was first described in 1997 [11, 12]. According to the latest nomenclature, CX3CL1 is the only representative of the CX3C (delta) subfamily [13]. Human CX3CL1 encoding gene is clustered on chromosome 16q13 [14]. Structurally, it differs from other chemokines by the presence of a motif encompassing three amino acid residues between two cysteine residues forming disulfide bonds which stabilize the tertiary structure of the molecule [11, 15]. Two isoforms of CX3CL1 occur in the body: soluble form (sCX3CL1) and membrane-anchored form (mCX3CL1) [11, 15]. The presence of two CX3CL1 forms in the body determines its unique role in the cytokine network. sCX3CL1 is a chemotactic factor for NK cells [16], T-lymphocytes [17], monocytes [18], and mastocytes [19]. Neutrophil binding and adhesion is influenced by mCX3CL1, which is particularly condensed within vascular endothelium cells (Figure 2). Therefore, the manifestation of the biological activity of the CX3CL1/CX3CR1 axis is most commonly observed in well-vascularized organs, such as the periosteum and periarticular tissues (e.g., the synovial membrane) [15]. In endothelial cells, mCX3CL1 functions as an adhesion molecule which facilitates the penetration of immune cells through the vascular endothelium regardless of the integrin-related mechanism [12, 13]. This extremely proinflammatory property facilitates a more rapid accumulation of immune system cells at the site of inflammation. Importantly, this property is unique as regards this group of molecules. To date, the participation of the CX3CL1/CX3CR1 axis has been confirmed in such pathologies as rheumatoid arthritis, systemic lupus erythematosus, atherosclerosis, CNS and spinal cord injury, and osteoarthritis (OA) [20–22].

Local CX3CL1 synthesis and expression is controlled by many factors, including inflammatory cytokines (IL-1*β*, interferon-*γ*, and TNF-*α*), lipopolysaccharide (LPS), or tissue oxygen tension (t*P*O_2_) [23, 24]. All the above-mentioned factors activate the network of intracellular transmitters and transcriptive factors, which results in an increased or reduced CX3CL1 synthesis [24].

Biological CX3CL1 activity may become apparent by interacting with CX3CR1 [25]. Structurally, CX3CR1 is one of the metabotropic receptors which are also called G-protein-coupled receptors (GPCR) or so-called 7-transmembrane proteins (7TM) [15]. The polypeptide chain includes 7 *α*-helical structures penetrating the whole thickness of the cell membrane. Therefore, three segments of the receptor may be distinguished: extracellular, transmembrane, and intracellular segments. Loops formed by the polypeptide chain externally bind ligands such as CX3CL1 [26] and CCL2 (CC chemokine ligand type 2) [27]. C-terminus and loops are located on the cytoplasmatic side which form the site where the heterotrimeric G*α*i protein is bound. CX3CR1, like other receptors for chemokines, is characterized by polymorphism which may define its variable affinity for CX3CL1 and present possibilities of selecting targeted therapies [28].

## 3. Possible Role of the CX3CL1/CX3CR1 Axis in Osteoclastogenesis

An experiment conducted by Koizumi et al. is one of the first studies which indicated the influence of the CX3CL1/CX3CR1 axis on the development of osteoporotic lesions [7]. 8-week-old mice were used to isolate osteoclast precursors (immature bone marrow cells, splenocytes, precursors of osteoclast line RAW 264.7), which were harvested from the tibia and the femur. Osteoblasts were also isolated from a similar site from 2-week-old mice. Mature osteoclasts (TRAP^+^ (tartrate-resistant acid phosphatase positive cells)) were obtained from osteoclast precursor cells after using RANKL (receptor activator for nuclear factor *κ* B ligand). All three types of cells (osteoclast precursors, mature osteoclasts, and osteoblasts) were then incubated with *α*-MEM (minimum essential medium eagle-alpha modification) and 10% FBS (fetal bovine serum), and further analyses were performed. First, CX3CR1 expression was assessed with the RT-PCR (reverse transcription-polymerase chain reaction) method in osteoclast precursor cells and mature osteoclasts. Marked overexpression and increased condensation of CX3CR1 were observed in precursor osteoclast cells. Such a tendency was not demonstrated in samples containing mature osteoclasts. A similar observation was made as regards samples of bones harvested from orthopedic patients, in which numerous CX3CR1^+^ cells (osteoclast precursors) were found. They tended to form CX3CL1^+^ osteoblast accumulations closer to bone surfaces. The authors suggested that the presence of CX3CR1 on osteoclast precursors indicated the effect of the CX3CL1/CX3CR1 axis on their differentiation into mature osteoclasts. It results from the fact that the use of anti-CX3CL1 mAB (mouse antibodies) markedly reduced the percentage of osteoclast precursors which differentiated into mature osteoclasts. Moreover, it was noted that those mature osteoclasts which expressed CX3CL1 formed numerous accumulations with CX3CR1^+^ cells (monocytes/macrophages) close to the bone surface. Over the first phase of the activation of sCX3CL1 immune cells, they are attracted towards the bony surface region including osteoclasts and other CX3CL1^+^ cells, such as vascular endothelium cells and dendritic and epithelial cells. During the second phase, CX3CR1^+^ macrophage/monocyte receptors are bound to osteoclast mCX3CL1 which leads to the activation of a local inflammatory response and the migration of monocytes/macrophages to the bone tissue.

Subsequent analyses by Koizumi et al. included a more detailed demonstration of the role of the CX3CL1/CX3CR1 axis in the development of osteoclasts via osteoblast binding. The addition of the CX3CL1 murine recombinant chemokine domain did not show an increased maturation of osteoclasts in the study samples. It is suggestive of the fact that the CX3CL1/CX3CR1 axis functions as a cofactor of the reaction of osteoclast maturation and not a direct stimulant of this reaction. Osteoblast-osteoclast precursor binding is necessary for the conversion of osteoclast precursors into mature osteoclasts, which occurs mainly via the CX3CL1/CX3CR1 axis. The activation of osteoclast precursor maturation into mature osteoclasts is managed by other signaling pathways (e.g., associated with RANKL). This factor may account for the fact that the addition of anti-CX3CL1 mAB produces distinct inhibition of osteoclast maturation, and adding recombined CX3CL1 does not increase the maturation (specific CX3CL1 concentration is necessary to cause binding with CX3CR1^+^ cells; additive effect is not observed above this concentration). Notably, an increased activation of the CCL2/CCR2 (CC chemokine receptor type 2) axis was observed in the described study apart from an increased condensation of CX3CR1 on osteoclast precursors after using RANKL. The biological manifestation of the CCL2/CCR2 signaling axis is generally similar to the CCL2/CX3CR1 and CX3CL1/CX3CR1 signaling pathways [29]. Interestingly, numerous studies confirmed that parathormone, whose increased concentrations are observed in the course of osteoporosis, influences the overexpression of the CCL2/CCR2 signaling axis, which probably indirectly influences an increased effectiveness of CX3CL1 binding to its dedicated receptors [30].

Another study conducted by Kikuta et al. [31] confirmed the participation of the CX3CL1/CX3CR1 axis in promoting osteoclastogenesis which is responsible for bone resorption and BMD reduction. First, the bone marrow was sampled from mice. Next, the researchers isolated three types of cells which are mostly responsible for osteoclastogenesis regulation: CD45^+^ CD11b^+^ cells (osteoblast precursors), CD45^+^ hematopoietic cells, and CXCR4^+^ CD45^−^ cells accounting for 28.4%, 58.8%, and 18.0% of sampled bone marrow cells, respectively. Basing on eliminative cultures, it was reported that the population of CXCR4^+^ CD45^−^ cells incubated with RANKL and M-CSF (macrophage colony-stimulating factor) demonstrated the most marked expression of factors influencing osteoclast maturation, including SDF-1 (stromal cell-derived factor 1), CXCL7 (CXC motif chemokine ligand 7), and CX3CL1. In order to confirm the role of CX3CL1 in osteoclastogenesis regulation, the following factors were added to RAW264.7 and bone marrow cells: antibodies against SDF-1, CXCL7, and CX3CL1. The reduction in the number and activity of osteoclasts was noted for all samples. Moreover, an influence of anti-SDF-1 and anti-CX3CL1 antibodies on osteoclast structure was observed—the colonies did not include large multinucleated mature osteoclasts but small and medium-sized less mature forms. The authors suggested that the CX3CL1/CX3CR1 axis may be responsible for the activation and fusion of osteoclasts in forming multinucleated mature structures.

The role of the CX3CL1/CX3CR1 axis in bone tissue resorption may be corroborated by a study by Kikuta et al. who used CX3CR1-EGFP^+^ knock-in mouse cells (enhanced green fluorescent protein-positive cells) in the assessment of the influence of active vitamin D (1,25-D) and its analogue—eldecalcitol (ELD)—on the expression of S1PR1 (sphingosine-1-phosphate receptor 1) [32]. CX3CR1-EGFP^+^ and CSF1R-EGFP^+^ (colony-stimulating factor 1 receptor) cells were recognized in the study model as the most precise model equivalent to osteoclast precursors. According to the authors, S1PR1 present on CX3CR1-EGFP^+^ cells promotes their migration from bones to the blood, which prevents the accumulation of osteoclast precursors in the bone tissue and catabolic activity. The second receptor for S1P (sphingosine-1-phosphate), S1PR2 (sphingosine-1-phosphate receptor 2), is S1PR1 antagonist [33] causing the reduction in the mobility of CX3CR1-EGFP^+^ cells which remain within the bone tissue. The activity of 1,25-D and ELD contributed to diminishing S1PR2 expression in CX3CR1-EGFP^+^ cells, which corroborated their therapeutic protective effect in the osseous tissue. No studies have been performed to analyze the direct correlation between S1P and the CX3CL1/CX3CR1 axis within the bone tissue. However, an antagonistic relation between those molecules may be observed in other tissues. A study conducted on cells, including those of the endocardium and endothelium, under *in vivo* conditions demonstrated that the use of S1P markedly decreased TNF-*α*-induced CX3CL1 expression [34]. It is a cellular anti-inflammatory/protective reaction which may largely influence the treatment of circulatory system pathologies. Presumably, a similar analogy between two cells also occurs within the bone tissue, which may be another confirmation of the influence of the CX3CR1/CX3CL1 axis on catabolic activity towards the bone tissue. However, it requires further research.

Mansell et al. [35] demonstrated a correlation between CX3CR1-EGFP^+^ cells (equivalent to osteoclast precursors) sampled from murine bone marrow and Debio0719, which is a lysophosphatidic acid (LPA) antagonist. LPA is a phospholipid whose activity is similar to growth factors for numerous types of cells, including nerve, epithelial, muscular cells, vascular endothelium cells, chondrocytes, osteoblasts, and osteoclasts [36]. The above-mentioned cells have 6 types of receptors for LPA, i.e., LPAR_1-6_ (lysophosphatidic acid receptor 1-6), which activate intracellular signaling pathways dependent on G*α*_i_, G*α*_12/13_, G*α*_q_, and G*α*_s_. The presence of LPAR_1_, LPAR_2_, LPAR_4_, LPAR_5_, and LPAR_6_ was noted in CX3CR1^+^ cells. According to the study, the most marked influence on the osseous tissue may be contributed to LPAR_1_ [37]. LPAR_1_ inhibition reduces the expression of osteoclast markers including adhesive *β*3 integrin and the tendency of osteoclast precursors towards maturation and fusion into multinucleated forms. It results in lowering the percentage of CX3CR1^+^ cells and mature osteoclasts at the bony surface because of their increased mobility to other tissues, which should be perceived as an anticatabolic activity. The reduced expression of *β*3 integrin in CX3CR1^+^ cells diminishes their potential of binding cell membranes, indirectly lowering the effectiveness of the CX3CL1/CX3CR1 pathway which has synergistic properties. The experiment may indicate a possible correlation between the CX3CL1/CX3CR1 axis and LPA/LPAR_1_ in the promotion of osteoclastogenesis processes, at the same time indicating the use of LPA antagonists to be a highly promising direction in the treatment of osteoporosis.

CX3CR1 expression on osteoclast precursors with the use of REV-ERBs (nuclear reverse erythroblastosis virus heme receptors) was investigated by Cho et al. [38]. REV-ERBs (REV-ERB*α* and REV-ERB*β*) are a group of transcriptional repressors, which regulate the expression of specific cell genes via binding nuclear compressor and histone deacetylase 3 complex. They are also responsible for the preservation of normal circadian rhythm and lipid conversion [6]. In this study, following REV-ERB supplementation, the culture of murine bone marrow-derived macrophages (BMMs) manifested a significantly lower expression for CX3CR1 (via inhibiting mRNA for the CX3CL1/CX3CR1 axis) than the control group which received the vector. It resulted in a distinct reduction in RANKL-induced osteoclastogenesis. The use of SR9009, which is a synthetic REV-ERB agonist, triggers a similar activity. Moreover, it leads to the shift in differentiating BMMs from inflammatory M1 macrophages to M2 macrophages which are characterized by anti-inflammatory activity. It is another example of the key role of the CX3CL1/CX3CR1 axis in the induction of inflammatory processes and osteoclastogenesis which are directly responsible for the development of osteoporotic lesions.

## 4. The Activity of the CX3CL1/CX3CR1 Axis in the Course of Osteoporosis

Basing on the available data, Chen et al. attempted to assess the concentrations of CX3CL1 in the blood serum (ELISA method) of patients with postmenopausal osteoporosis (*n* = 53, study group, PMOP) compared to healthy postmenopausal patients (*n* = 51, PMNOP) and healthy premenopausal patients (*n* = 50)—2 control groups [6]. The obtained data were correlated with the markers of osteoporosis development, i.e., the level of bone turnover factors and inflammatory factors in blood serum like TRACP-5b (tartrate-resistant acid phosphatase 5b), NTx (cross-linked N-telopeptides of type I collagen), IL-1*β*, IL-6, and oestrogen-2 (E2). Additionally, all the patients had undergone densitometry (DXA) of the lumbosacral segment (L-S) of the spine, femoral neck, the greater trochanter, and Ward's triangle. They also completed the Visual Analogue Scale (VAS) and Oswestry Disability Index (ODI) questionnaires. No significant differences regarding age were reported between the groups of postmenopausal women. All 3 groups did not differ in terms of Body Mass Index (BMI). The obtained results explicitly indicated the correlation between CX3CL1 concentration in blood serum and the degree of osteoporosis development. The comparison of CX3CL1 concentrations in PMOP patients compared with PMNOP and premenopausal patients showed the respective results of 139.8 ± 44.3 pg/mL, 116.5 ± 23.1 pg/mL, and 109.7 ± 19.4 pg/mL with *p* < 0.05. As expected, DXA examination showed that BMD was significantly lower in PMOP patients in all locations. It increased in the PMNOP group and reached the highest values in premenopausal women. The values were inversely proportional to CX3CL1 concentrations in blood serum. The concentrations of bone turnover markers (TRACP-5b: *r* = 0.341, *p* = 0.012; NTx: *r* = 0.364, *p* = 0.007) and inflammatory factors (IL-1*β*: *r* = 0.396, *p* = 0.003; IL-6: *r* = 0.355, *p* = 0.009) increased proportionally to increasing CX3CL1 concentrations. The authors express an opinion that this study is promising in terms of viewing CX3CL1 as a marker of the exacerbation of osteoporotic lesions and indicate a possibility of employing this correlation in screening tests. It is also emphasized that longitudinal studies and additional research should be conducted in larger groups of patients. From the viewpoint of the present authors, it would be beneficial to obtain osteoclast cell lines from PMOP and PMNOP patients for cultures and assessment of CX3CR1 expression. It is advisable to assess the concentrations of growth factors responsible for BMD increase and correlating negatively with the CX3CL1/CX3CR1 axis, such as VEGF or TGF-*β* [39, 40]. It might constitute an even stronger confirmation of the role of the CX3CL1/CX3CR1 axis in the pathogenesis of osteoporosis and indicate CX3CL1 as the best immunological marker of the risk of osteoporosis or prognosis in its course.

## 5. Conclusions and Future Perspectives

Osteoporosis is a civilization disease which is still challenging for contemporary medicine not only in terms of treatment but also prophylaxis. It is closely related to decreasing the quality of life and numerous complications resulting from BMD reduction, including fractures and accelerated progress of osteoarthritis [41, 42]. Osteoporotic lesions also occur in numerous articular pathologies, such as rheumatoid arthritis or hemophilic arthropathy [43–45]. It results in very serious secondary consequences comprising the limitation or complete immobilization of patients, cardiovascular, infectious, or thrombotic complications [46]. Therefore, osteoporosis should be viewed as a disease which increases mortality, especially in elderly patients.

It is assumed that the causes of osteoporosis are commonly known (endocrine disorders, neoplastic diseases, excessive alcohol consumption, smoking, and malnutrition), but attempts at assessing its etiology basing on the immune system are relatively rare. Another difficulty is connected with the location of immune processes in the dependency network with other causes of the disease. The resultant question is whether the activity of inflammatory/catabolic factors is caused by such factors as endocrine changes or endocrine changes are triggered by primary disturbance of balance between inflammatory and anti-inflammatory cytokines.

It is difficult to indicate whether the role of immune processes constitutes the primary or secondary reason for the development of osteoporotic lesions. However, the expression of signaling axes associated with inflammatory factors, such as the CX3CL1/CX3CR1 axis, is certainly increased in osteoporosis (Table 1). The present study includes convincing evidence of a significant role of this axis in processes leading to BMD reduction. Osteoclast precursors are characterized by an increased condensation of CX3CR1 on the surface, which after binding mCX3CL1 play a principal role in osteoclast maturation (development of multinucleated forms) and binding them to the surface of the bone tissue, where they may manifest their catabolic activity. The role of sCX3CL1 consists in attracting not only osteoclast precursors to the osseous tissue but also other immune system cells, which form numerous accumulations activated on the bony surface (via CX3CR1), promoting the development of inflammation and production of other inflammatory cytokines (i.e., TNF-*α*, IL-1*β*, and IL-6). The CX3CL1/CX3CR1 axis also plays a key role in the maturation of osteoclast precursors via binding osteoblasts. The overexpression of this axis may recruit considerable quantities of osteoblasts bound with the bony surface to osteoclastogenesis which shifts osseous tissue metabolism towards catabolism. All the observations were noted on murine models, both *in vitro* and *in vivo*, especially through the analysis of the use of specific anti-CX3CL1 antibodies. In each case, after using the antibody, cellular colonies did not present mature multinucleated osteoclasts, but only smaller intermediate forms without chemotaxis towards the bony surface.

Apart from the direct correlation between the CX3CL1/CX3CR1 axis and activation/maturation of osteoclasts and induction of inflammatory processes, the CX3CL1/CX3CR1 axis may indirectly reduce BMD in a more subtle manner. Highly possible suppressive S1P activity on TNF-*α*-induced CX3CL1 expression may serve as an example, which contributes to a higher degree of the migration of osteoclast precursors beyond the osseous tissue. Therefore, the stimulation of the S1P/S1PR1 axis sensitive to vitamin D3 and its analogues presents antagonistic properties to the CX3CL1/CX3CR1 axis. Reducing vitamin D3 concentrations in blood serum reported in the course of osteoporosis may contribute to unblocking and increasing the expression of the CX3CL1/CX3CR1 signaling pathway, which, according to previous research, stimulates the process of osteoclastogenesis and further BMD reduction. Notably, the process of accumulating osteoclast precursors in the osseous tissue and its maturation are not only influenced by inflammatory factors but also molecules with growth potential, such as LPA. The LPA/LPAR_1_ pathway stimulates the osteoclastogenesis of CX3CR1^+^ cells and the expression of osteoclast markers, e.g., *β*3 integrin. The marker demonstrates synergy with the CX3CL1/CX3CR1 axis as regards increasing the tendency towards binding cell membranes of various cell types and more effective osteoclast maturation. Inhibiting LPAR_1_ (e.g., by Debio0719) results in weakening the CX3CL1/CX3CR1 signaling pathway which again corroborates the participation of this axis in the development of osteoporosis. Inhibiting the process of osteoclastogenesis through blocking mRNA for the CX3CL1/CX3CR1 axis in osteoclast precursors (using REV-ERBs or SR9009) is another strong piece of evidence of its participation in immune processes leading to BMD reduction.

In spite of the availability of promising and rather unambiguous data obtained from murine models, only one study concerning the correlation between CX3CL1 concentration in blood serum and osteoporosis-related markers has been conducted in humans. The study also indicated a high probability of CX3CL1 contribution to the degree of the intensification of osteoporotic lesions. The highest concentrations of CX3CL1 were observed in PMOP patients, and it was directly proportional to increased concentrations of bone turnover and inflammatory factors in blood serum (TRACP-5b, NTx, IL-1*β*, and IL-6). In those patients, the lowest BMD values were observed in locations which are typically prone to osteoporotic fractures (e.g., proximal femur, L-S spinal segment) measured with DXA. The remaining groups of patients (PMNOP and healthy premenopausal women) had significantly lower concentrations of CX3CL1 in blood serum which correlated with lower concentrations of bone turnover markers and a higher BMD in DXA examination.

Therefore, the role of the CX3CL1/CX3CR1 axis in the development and exacerbation of osteoporotic lesions seems very well documented at present. Several disadvantages of the research may be listed: analyses conducted mainly on murine models and not in humans, small study group size, and lack of longitudinal studies or fully standardized laboratory tests. Few research papers have been published on the possible role of the CX3CL1/CX3CR1 axis in promoting the process of osteoclastogenesis, although it was first described in 2009 [7]. Regarding the fact that acting against the CX3CL1/CX3CR1 axis is a potential target of immune combination treatment in osteoporosis, the number of available papers tackling the topic is certainly insufficient. The results of the present study necessitate asking new questions as regards the role of the CX3CL1 axis. It would be of great value to assess CX3CR1 condensation not only on osteoclast precursors and osteoblasts but also on cells which naturally surround the osseous tissue, i.e., fibroblasts or vascular endothelium, which activity may trigger the shift of inflammation from the circulatory system (systemic) to the bone tissue (local). The network of blood vessels supplying the bone also plays a considerable role in osteoporosis. The observation of the correlation of the concentration of VEGF and other growth factors in the vascular endothelium compared to CX3CL1 concentrations in blood serum and DXA results might broaden the knowledge regarding the role of CX3CL1/CX3CR1 as an axis which not only activates osteoclastogenesis but also affects on local perfusion and bone nourishment [47].

The research conducted by Cho et al. [38] concerning the effect of the CX3CL1/CX3CR1 axis on mRNA expression leads to the question whether gene polymorphism for CX3CL1/CX3CR1 contributes to osteoclastogenesis and a possible risk of developing osteoporosis in the future. Determining such a correlation might lead to a situation where a simple genetic test of the blood would indicate risk groups in which appropriate prophylaxis and follow-up examinations might be introduced.

Osteoporosis is a multifactorial disease, so moderate optimism is recommended as regards the decisive role of the CX3CL1/CX3CR1 axis in the pathophysiology of this disease. The possibility of treatment based on specific antibodies is not expected to be the sole therapeutic method. Most probably, it may become a valuable element of combination treatment. Primary or secondary overexpression of the CX3CL1/CX3CR1 axis is only one of the numerous possible causes of osteoporosis, but it seems to play a central role in the context of immunity-related causes. Therefore, it seems justified to continue research which would precisely determine its role in the metabolism of the bone tissue as one of the most promising targets in osteoporosis therapy.

## Figures and Tables

**Table 1:** The potential role of the CX3CL1/CX3CR1 signaling axis in osteoclastogenesis. OCs: osteoclasts; OBs: osteoblasts; BMCs: bone marrow cells; CD: cluster of differentiation; CXCR4: CXC chemokine receptor type 4; BMD: bone mineral density; CSF1R: colony-stimulating factor 1 receptor; EGFP: enhanced green fluorescent protein; CX3CR1: CX3C chemokine receptor 1; LPA: lysophosphatidic acid; LPAR1: lysophosphatidic acid receptor type 1; BMMs: bone marrow macrophages; CX3CL1: CX3C motif chemokine ligand 1; PMOP: postmenopausal osteoporosis patients; PMNOP: healthy postmenopausal patients; TRACP-5b: tartrate-resistant acid phosphatase 5b; NTx: cross-linked N-telopeptides of type I collagen; IL-1*β*: interleukin 1 beta; IL-6: interleukin 6.

Study	Material	CX3CL1/CX3CR1 axis role
Koizumi et al.	(i) Mouse precursors of OCs (Toyama, Japan) (ii) Mouse OBs (Toyama, Japan) (iii) Mature OCs (Wako Pure Chemical) (iv) Bone samples of orthopedic patients (University of Toyama, Toyama, Japan)	(i) Stimulation of differentiation of OC precursors into mature OCs (ii) Activation of local inflammatory reaction and migration of monocytes/macrophages to bone tissue (iii) Cofactor for the OC maturation and OB binding

Goto et al.	(i) Mouse BMCs (CD45 CD11b^+^, CD45^+^ hematopoietic cells, CXCR4^+^ CD45^−^, Shizuoka, Japan)	(i) Promotion of osteoclastogenesis (ii) Bone resorption and BMD reduction (iii) Activation and fusion of OCs in the formation of multijugular forms

Kikuta et al.	(i) CD11b^+^ cells from C57BL/6 mice (CREA Japan) (ii) BMCs from CSF1R-EGFP^+^ transgenic mice (Jackson Laboratory) (iii) BMCs from CX3CR1-EGFP^+^ knock-in mice (Jackson Laboratory) (iv) Mouse RAW264.7 cells (Chugai Pharmaceuticals Co., Ltd.)	(i) Stimulation of mobility of CX3CR1-EGFP^+^ cells near bone tissue

David et al.	(i) BMCs from CX3CR1-EGFP^+^ knock-in mice (Jackson Laboratory)	(i) Potential correlation with LPA/LPAR1 in promoting osteoclastogenesis process

Song et al.	(i) BMMs from C57BL/6 mice (CREA Japan)	(i) Induction of inflammatory processes and osteoclastogenesis

Chen et al.	Concentration of CX3CL1 in blood serum in group of (i) 53 PMOP (ii) 51 PMNOP (iii) 50 healthy premenopausal patients	(i) Increasing the concentration of bone turnover markers (TRACP-5b, NTx) and inflammatory factors (IL-1*β*, IL-6) in blood serum (ii) Potential marker and screening test of the exacerbation of osteoporotic lesions

## References

[1] Wein M. N. (2017). Parathyroid hormone signaling in osteocytes. *JBMR Plus*.

[2] Matsuo K., Irie N. (2008). Osteoclast-osteoblast communication. *Archives of Biochemistry and Biophysics*.

[3] Kenkre J. S., Bassett J. (2018). The bone remodelling cycle. *Annals of Clinical Biochemistry: International Journal of Laboratory Medicine*.

[4] Riancho J. A. (2015). Epigenetics of osteoporosis: critical analysis of epigenetic epidemiology studies. *Current Genomics*.

[5] Azizieh F., Raghupathy R., Shehab D., Al-Jarallah K., Gupta R. (2017). Cytokine profiles in osteoporosis suggest a proresorptive bias. *Menopause*.

[6] Chen Y. D., Huang C. Y., Liu H. Y. (2016). Serum CX3CL1/fractalkine concentrations are positively associated with disease severity in postmenopausal osteoporotic patients. *British Journal of Biomedical Science*.

[7] Koizumi K., Saitoh Y., Minami T. (2009). Role of CX3CL1/fractalkine in osteoclast differentiation and bone resorption. *Journal of Immunology*.

[8] Palomino D. C., Marti L. C. (2015). Chemokines and immunity. *Einstein (São Paulo)*.

[9] Liu W., Jiang L., Bian C. (2016). Role of CX3CL1 in diseases. *Archivum Immunologiae et Therapiae Experimentalis*.

[10] Bazan J. F., Bacon K. B., Hardiman G. (1997). A new class of membrane-bound chemokine with a CX3C motif. *Nature*.

[11] Pan Y., Lloyd C., Zhou H. (1997). Neurotactin, a membrane-anchored chemokine upregulated in brain inflammation. *Nature*.

[12] Zlotnik A., Yoshie O. (2012). The chemokine superfamily revisited. *Immunity*.

[13] Nomiyama H., Imai T., Kusuda J., Miura R., Callen D. F., Yoshie O. (1998). Human chemokines fractalkine (SCYD1), MDC (SCYA22) and TARC (SCYA17) are clustered on chromosome 16q13^1^. *Cytogenetic and Genome Research*.

[14] Kim K. W., Vallon-Eberhard A., Zigmond E. (2011). In vivo structure/function and expression analysis of the CX3C chemokine fractalkine. *Blood*.

[15] Hamann I., Unterwalder N., Cardona A. E. (May). Analyses of phenotypic and functional characteristics of CX3CR1-expressing natural killer cells. *Immunology*.

[16] Mionnet C., Buatois V., Kanda A. (2010). CX3CR1 is required for airway inflammation by promoting T helper cell survival and maintenance in inflamed lung. *Nature Medicine*.

[17] Ancuta P., Rao R., Moses A. (2003). Fractalkine preferentially mediates arrest and migration of CD16+monocytes. *The Journal of Experimental Medicine*.

[18] Papadopoulos E. J., Fitzhugh D. J., Tkaczyk C. (2000). Mast cells migrate, but do not degranulate, in response to fractalkine, a membrane-bound chemokine expressed constitutively in diverse cells of the skin. *European Journal of Immunology*.

[19] Wojdasiewicz P., Poniatowski L. A., Kotela A., Deszczyński J., Kotela I., Szukiewicz D. (2014). The chemokine CX3CL1 (fractalkine) and its receptor CX3CR1: occurrence and potential role in osteoarthritis. *Archivum Immunologiae et Therapiae Experimentalis (Warsz)*.

[20] Muñoz L. M., Holgado B. L., Martínez-A C., Rodríguez-Frade J. M., Mellado M. (2012). Chemokine receptor oligomerization: a further step toward chemokine function. *Immunology Letters*.

[21] Mellado M., Martínez-A C., Rodríguez-Frade J. M. (2002). Analysis of G-protein-coupled receptor dimerization following chemokine signaling. *Methods*.

[22] Zujovic V., Benavides J., Vigé X., Carter C., Taupin V. (2000). Fractalkine modulates TNF-*α* secretion and neurotoxicity induced by microglial activation. *Glia*.

[23] Cambien B., Pomeranz M., Schmid-Antomarchi H. (2001). Signal transduction pathways involved in soluble fractalkine-induced monocytic cell adhesion. *Blood*.

[24] Imai T., Hieshima K., Haskell C. (1997). Identification and molecular characterization of fractalkine receptor CX3CR1, which mediates both leukocyte migration and adhesion. *Cell*.

[25] Mizoue L. S., Bazan J. F., Johnson E. C., Handel T. M. (1999). Solution structure and dynamics of the CX3C chemokine domain of fractalkine and its interaction with an N-terminal fragment of CX3CR1†^,^‡. *Biochemistry*.

[26] Nakayama T., Watanabe Y., Oiso N. (2010). Eotaxin-3/CC chemokine ligand 26 is a functional ligand for CX3CR1. *Journal of Immunology*.

[27] Niessner A., Marculescu R., Haschemi A. (2005). Opposite effects of CX3CR1 receptor polymorphisms V249I and T280M on the development of acute coronary syndrome. *Thrombosis and Haemostasis*.

[28] Käufer C., Chhatbar C., Bröer S. (2018). Chemokine receptors CCR2 and CX3CR1 regulate viral encephalitis-induced hippocampal damage but not seizures. *Proceedings of the National Academy of Sciences of the United States of America*.

[29] Li X., Qin L., Bergenstock M., Bevelock L. M., Novack D. V., Partridge N. C. (2007). Parathyroid hormone stimulates osteoblastic expression of MCP-1 to recruit and increase the fusion of pre/osteoclasts. *Journal of Biological Chemistry*.

[30] Goto Y., Aoyama M., Sekiya T. (2016). CXCR4+ CD45- cells are niche forming for osteoclastogenesis via the SDF-1, CXCL7, and CX3CL1 signaling pathways in bone marrow. *Stem Cells*.

[31] Kikuta J., Kawamura S., Okiji F. (2013). Sphingosine-1-phosphate-mediated osteoclast precursor monocyte migration is a critical point of control in antibone-resorptive action of active vitamin D. *Proceedings of the National Academy of Sciences of the United States of America*.

[32] Yang J., Yang L., Tian L., Ji X., Yang L., Li L. (2018). Sphingosine 1-phosphate (S1P)/S1P receptor_2/3_ axis promotes inflammatory M1 polarization of bone marrow-derived monocyte/macrophage via G(I)_i/o_/PI3K/JNK pathway. *Cellular Physiology and Biochemistry*.

[33] Ahn S. Y., Cho C. H., Park K. G. (2004). Tumor necrosis factor-*α* induces fractalkine expression preferentially in arterial endothelial cells and mithramycin A suppresses TNF-*α*-induced fractalkine expression. *The American Journal of Pathology*.

[34] David M., Machuca-Gayet I., Kikuta J. (2014). Lysophosphatidic acid receptor type 1 (LPA1) plays a functional role in osteoclast differentiation and bone resorption activity. *The Journal of Biological Chemistry*.

[35] Mansell J. P., Nowghani M., Pabbruwe M., Paterson I. C., Smith A. J., Blom A. W. (2011). Lysophosphatidic acid and calcitriol co-operate to promote human osteoblastogenesis: requirement of albumin-bound LPA. *Prostaglandins & Other Lipid Mediators*.

[36] Gennero I., Laurencin-Dalicieux S., Conte-Auriol F. (2011). Absence of the lysophosphatidic acid receptor LPA1 results in abnormal bone development and decreased bone mass. *Bone*.

[37] Song C., Tan P., Zhang Z. (2018). REV-ERB agonism suppresses osteoclastogenesis and prevents ovariectomy-induced bone loss partially via FABP4 upregulation. *The FASEB Journal*.

[38] Cho H., Zhao X., Hatori M. (2012). Regulation of circadian behaviour and metabolism by REV-ERB-*α* and REV-ERB-*β*. *Nature*.

[39] Sciumè G., Soriani A., Piccoli M., Frati L., Santoni A., Bernardini G. (2010). CX3CR1/CX3CL1 axis negatively controls glioma cell invasion and is modulated by transforming growth factor-*β*1. *Neuro-Oncology*.

[40] Johnell O., Kanis J. (2005). Epidemiology of osteoporotic fractures. *Osteoporosis International*.

[41] Yaacobi E., Sanchez D., Maniar H., Horwitz D. S. (2017). Surgical treatment of osteoporotic fractures: an update on the principles of management. *Injury*.

[42] Kotela A., Lorkowski J., Żbikowski P., Ambroziak P., Kucharzewski M., Kotela I. (2015). Total ankle arthroplasty in patients with inherited bleeding disorders. *Haemophilia*.

[43] Kotela I., Zbikowski P., Ambroziak P. (2014). Total elbow arthroplasty in patient with severe von Willebrand disease. *Haemophilia*.

[44] Wojdasiewicz P., Poniatowski Ł. A., Nauman P. (2018). Cytokines in the pathogenesis of hemophilic arthropathy. *Cytokine & Growth Factor Reviews*.

[45] Ensrud K. E., Crandall C. J. (2017). Osteoporosis. *Annals of Internal Medicine*.

[46] Coughlan T., Dockery F. (2014). Osteoporosis and fracture risk in older people. *Clinical Medicine (London, England)*.

[47] Lu P., Li L., Kuno K. (2008). Protective roles of the fractalkine/CX3CL1-CX3CR1 interactions in alkali-induced corneal neovascularization through enhanced antiangiogenic factor expression. *Journal of Immunology*.

